# Plasma Gut Microbe-Derived Metabolites Associated with Peripheral Artery Disease and Major Adverse Cardiac Events

**DOI:** 10.3390/microorganisms10102065

**Published:** 2022-10-19

**Authors:** Karen J. Ho, Joel L. Ramirez, Rohan Kulkarni, Katharine G. Harris, Irene Helenowski, Liqun Xiong, C. Keith Ozaki, S. Marlene Grenon

**Affiliations:** 1 Division of Vascular Surgery, Northwestern University Feinberg School of Medicine, Chicago, IL 60611, USA; 2 Division of Vascular & Endovascular Surgery, University of California San Francisco, San Francisco, CA 94143, USA; 3 Division of Vascular Surgery, University of Pittsburgh, Pittsburgh, PA 15213, USA; 4 Department of Biology, Franklin College, Franklin, IN 46131, USA; 5 Department of Preventive Medicine, Northwestern University Feinberg School of Medicine, Chicago, IL 60611, USA; 6 Division of Vascular and Endovascular Surgery, Brigham and Women’s Hospital, Boston, MA 02115, USA

**Keywords:** peripheral artery disease, ankle-brachial index, microbiome

## Abstract

Cardiovascular diseases are associated with gut dysbiosis, but the role of microbe-derived metabolites as biomarkers or modulators of cardiovascular disease are not well understood. This is a targeted metabolomics study to investigate the association of nine microbe-derived metabolites with lower extremity peripheral artery disease (PAD), a form of atherosclerosis, and major adverse cardiac events (MACE). The study cohort consists of individuals with intermittent claudication and ankle-brachial index (ABI) < 0.9 (N = 119) and controls without clinically-apparent atherosclerosis (N = 37). The primary endpoint was MACE, a composite endpoint of myocardial infarction, coronary revascularization, stroke, transient ischemic attack, or cardiac-related death. Plasma metabolite concentrations differed significantly between the PAD and control groups. After adjustment for traditional atherosclerosis risk factors, kynurenine, hippuric acid, indole-3-propionic acid (IPA), and indole-3-aldehyde (I3A) concentrations were negatively associated with PAD, whereas indoxyl sulfate and 3-hydroxyanthranilic acid were positively associated. Hippuric acid, IPA, and I3A correlated with ABI, a surrogate for atherosclerotic disease burden. Those in the highest I3A concentration quartile had significantly improved freedom from MACE during follow-up compared to those in the lowest quartile. This study identifies specific indole- and phenyl-derived species impacted by gut microbial metabolic pathways that could represent novel microbiome-related biomarkers of PAD.

## 1. Introduction

Multiple lines of evidence point towards a modulatory role of the gut microbiome in atherosclerosis. Germ-free mice with a genetic predisposition for atherosclerosis (apolipoprotein E deficiency) that were fed a low fat diet had less atherosclerotic plaques compared to conventionally-raised mice [1]; microbial producers of butyrate, a short-chain fatty acid, have a protective effect against atherosclerosis [2]; and trimethylamine-N-oxide, a derivative of the microbial metabolite trimethylamine, is both atherogenic in animal models and associated with cardiovascular events in humans [3,4,5,6]. Patients with symptomatic carotid stenosis requiring revascularization have been found to have altered microbiota compared to age- and gender-matched controls [7]. Other cardiovascular diseases have also recently been associated with gut dysbiosis, including coronary artery disease [8,9,10], hypertension [11,12,13], stroke [14,15,16,17], heart failure [18,19,20].

While metabolomic phenotyping allows for detailed documentation of the global functional output of the gut microbiome through qualitative and quantitative analyses of the mammalian–microbial co-metabolome [21], the functional role of most of the commensal metabolome in cardiovascular disease is not well understood. We previously identified correlations between systemic concentration of microbe-derived indole- and phenyl-derived metabolites [22] and advanced atherosclerosis in a heterogeneous population of vascular surgery patients undergoing procedures for advanced atherosclerosis [23]. The cohort with advanced atherosclerosis had either carotid or lower extremity peripheral artery disease (PAD). Given the heterogeneity in the clinical presentation and severity of atherosclerosis in the previous study, the current study is focused only on people with intermittent claudication, a form of PAD in which pain in the legs occurs with exercise and is relieved by rest (Rutherford category 1–3) [24]. The purpose of this study is to investigate the ability of these selected microbe-derived metabolites to discriminate between a well-characterized cohort of participants with intermittent claudication and non-PAD controls.

## 2. Materials and Methods

### 2.1. Study Population

The PAD cohort consists of participants in the placebo arm of the OMEGA PAD trials at the San Francisco VA Medical Center [25,26,27] between 2011 and 2016, all of whom were claudicants (Rutherford category 1–3) with PAD confirmed by the ankle-brachial index (ABI) < 0.9 and were age ≥ 50 years. Exclusion criteria in the OMEGA PAD trials were severe hepatic (Child–Pugh ≥ B) or renal (creatinine ≥ 2 mg/dL) disease, chronic limb-threatening ischemia, or nonvascular inflammatory disease. Patients were also excluded if they had a severe acute illness within 30 days or were taking immunosuppressive medications or steroids. An additional cohort of patients with no history of PAD, no clinical atherosclerotic disease (coronary artery or cerebrovascular disease), and ABI > 0.9 served as non-PAD controls. Demographic and clinical data were collected prospectively. Blood was drawn at the baseline visit and plasma was immediately prepared, aliquoted, and stored at −80 °C. Plasma aliquots had not been thawed before this study. The investigator-initiated protocol was approved by the University of California, San Francisco Committee on Human Research as well as the San Francisco Veterans Affairs Research and Development Office. Written informed consent was obtained. The OMEGA PAD trials were registered with ClinicalTrials.gov (unique identifiers: NCT01310270 and NCT01979874).

### 2.2. Variables

Sociodemographic variables included sex, age, and race. Inflammatory markers including high-sensitivity C-reactive protein (CRP) were measured as previously described [25,26]. Estimated glomerular filtration rate (eGFR) was calculated using the Modification of Diet in Renal Disease (MDRD) study equation [28]. Past medical history and medication use in both the PAD and non-PAD groups were collected at the time of original study enrollment. Smoking was defined as current or prior tobacco use. Bilateral resting ABI was measured at the baseline visit in the following manner. Systolic blood pressures of the brachial, posterior tibial, and dorsalis pedis arteries were measured bilaterally. For each lower extremity, the highest systolic pressure of the 2 pedal pulses was divided by the highest systolic pressure of the 2 brachial arteries. The ABI was classified as “incompressible” if ≥ 1.3. The omega-3 index is a measure of omega-3 fatty acids, eicosapentaenoic acid (EPA), and docosahexaenoic acid (DHA) in red blood cell membranes and is recognized as an independent biomarker for cardiovascular health and disease [29]. The omega-3 index was measured using previously published methods [30].

### 2.3. Endpoints

The primary endpoint was major adverse cardiovascular event (MACE), defined as a composite endpoint of myocardial infarction (MI), coronary revascularization, stroke, transient ischemic attack, or cardiac-related death [27]. Data on the date of these events were abstracted retrospectively from the electronic medical record. MI was defined according to the American Heart Association universal definition of acute MI [31]. Stroke was defined as any new embolic, thrombotic, or hemorrhagic cerebrovascular event with neurologic deficits that persisted for at least 24 hours, as defined by an attending neurologist.

### 2.4. Detection and Quantification of Metabolites by High-Performance Liquid Chromatography–Tandem Mass Spectrometry (LC-MS/MS)

Plasma concentrations of metabolites (*p*-cresyl sulfate [PCS], hippuric acid [HA], indole-3-propionic acid [IPA], tryptophan [TRP], kynurenine [KYN], indoxyl sulfate [IS], serotonin, indole-3-aldehyde [I3A], and 3-hydroxyanthranilic acid [HAA]) in 50 μL plasma were measured as previously described [23] in a 96-well plate format.

### 2.5. Statistical Analysis

Continuous variables are reported as median values with interquartile ranges and were compared using the Wilcoxon signed rank test or the Student’s t-test based on the normality of distribution. Associations between continuous variables were evaluated by Spearman rank correlation. Categorical variables are reported as frequencies and percentages and compared using Chi-squared or Fisher’s exact testing. Metabolite concentrations were natural log (ln) transformed to reduce skewness before regression analyses. A *p* value < 0.05 was considered statistically significant. All statistical analyses were performed using SAS 9.4 (SAS Institute, Cary, NC, USA) and GraphPad Prism 7 (GraphPad Software, Inc., La Jolla, CA, USA).

## 3. Results

### 3.1. Study Population

Baseline characteristics of the claudication (N = 119) and non-PAD cohorts (N = 37) are shown in Table 1. The claudication cohort had a greater prevalence of coronary artery disease, hypertension, hyperlipidemia, smoking, and history of prior MACE compared to the control cohort. As anticipated, the claudication cohort also had more frequent use of aspirin and statin medication and worse ABI. Interestingly, median low-density lipoprotein cholesterol concentration was lower in the claudication group, likely attributable to statin use. The follow-up period ranged from 2.5 to 3.2 years, with a median of 2.9 years (interquartile range 2.3–3.6)

Claudicants had significantly higher baseline plasma concentrations of inflammatory cytokines IL-6, ICAM, and TNF-α and a significantly lower omega-3 index compared to non-PAD controls. There were also significant unadjusted differences in the concentrations of the microbe-derived metabolites of interest, as shown in Table 1. Claudicants had significantly higher plasma concentrations of IS, HAA, and PCS and lower baseline plasma concentrations of serotonin, KYN, TRP, IPA, I3A, and HA than non-PAD controls. The KYN/TRP ratio, a surrogate for indole-2,3-deoxygenase (IDO1) and tryptophan-2,3- deoxygenase (TDO) activity [32], was not observed to be significantly different between the groups. After adjusting the metabolites for sex and traditional risk factors for atherosclerosis (smoking, hypertension, diabetes, and hyperlipidemia) (Table 2), KYN (OR 0.22; 95% CI 0.086–0.57; *p* = 0.002), HA (OR 0.42; 95% CI, 0.24–0.72; *p* = 0.002), IPA (OR 0.36; 95% CI, 0.21–0.61; *p* = 0.0002), and I3A (OR 0.11; 95% CI, 0.03–0.35; *p* = 0.0002) remained significantly negatively associated with claudication, while IS (OR 1.8; 95% CI, 1.11–3.00); *p* = 0.02) and HAA (OR 4.44; 95% CI 2.6–7.5; *p* < 0.0001) correlated positively with claudication.

### 3.2. Correlation between Metabolites and ABI

**Figure 1:**
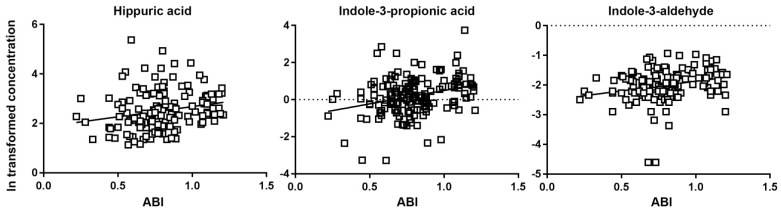
Correlations between ln-transformed metabolite concentrations and ankle-brachial index (ABI). Only correlations with *p* < 0.05 are shown. Each line represents the linear regression of each metabolite on ABI. Spearman coefficients and *p* values are provided in the text.

PAD is associated with prevalent cardiovascular disease and adverse cardiovascular disease risk factor profiles [33,34,35]. Prospective studies using ABI have shown that a low ABI predicts both cardiovascular and all-cause mortality in people with and without existing clinical coronary artery disease [35,36,37,38,39,40,41,42,43,44,45,46]. Therefore, we examined the Spearman correlation between each metabolite and ABI. As shown in Figure 1, HA (Spearman r = 0.29; *p* = 0.0004), IPA (Spearman r = 0.27; *p* = 0.001), and I3A (Spearman r = 0.3; *p* = 0.0004) had modest but statistically significant positive correlations with ABI.

### 3.3. Relationship between Metabolites and MACE

**Figure 2:**
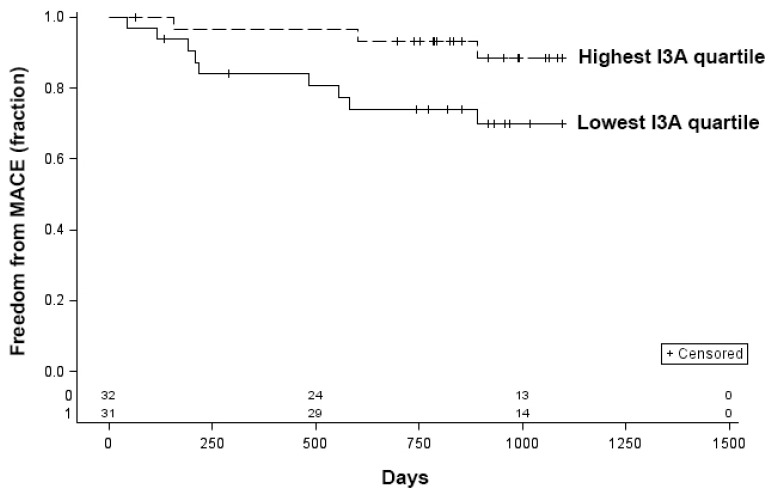
Kaplan–Meier curves for freedom from MACE for patients in the lowest and highest quartiles of indole-3-aldehyde (I3A) concentration. *p* = 0.045, log-rank.

Data on MACE were available on 139 participants. Of these, 28 (20.1%) patients experienced a MACE during the follow-up period. Univariate analysis comparing patients who had a MACE with those without MACE is shown in Table 3. Patients who experienced MACE were more likely to have coronary artery disease (*p* = 0.0002) and a decreased ABI (*p* = 0.01) compared to those who did not have a MACE. In addition, baseline unadjusted KYN (*p* = 0.02), TRP (*p* = 0.003), and I3A (*p* = 0.02) were significantly associated with MACE on univariate analysis. When patients were stratified into groups by using the quartiles of metabolite concentrations, patients in the highest I3A quartile had significantly improved freedom from MACE compared to patients in the lowest I3A quartile in a Kaplan–Meier analysis (*p* = 0.045, log-rank) (Figure 2). Differences in freedom from MACE based on the lowest and highest quartiles of KYN and TRP did not reach statistical significance (KYN, *p* = 0.45; TRP, *p* = 0.38; log-rank).

## 4. Discussion

We demonstrate that baseline plasma concentrations of multiple gut microbe-derived indole- and phenyl-derived metabolites (KYN, TRP, IPA, I3A, IS, HAA, and HA) are associated with presence of lower extremity PAD and with MACE. Specifically, after adjustment for traditional risk factors for atherosclerosis, KYN, HA, IPA, I3A, and IS are associated with decreased risk of claudication, while IS and HAA are associated with an increased risk. Furthermore, there was a significant positive association between baseline KYN, TRP, and I3A levels and MACE.

These findings are largely in concordance with our previous study describing an association between indole- and phenyl-derived metabolites that are either exclusively or partly produced via microbial metabolic pathways and advanced atherosclerosis [23]. In this study, we observed that claudicants had significantly lower IPA and I3A levels compared to non-PAD controls (OR 0.36 and OR 0.11, respectively, *p* = 0.0002 for both). However, new associations were made between IS, HAA, HA, and PAD, while previous observations linking TRP and the KYN/TRP ratio to advanced atherosclerosis [23] were not seen in the current study.

Like the previous study [23], we also observed modest yet statistically significant positive correlations between HA, IPA, and I3A and ABI. A diagnosis of PAD can be made clinically or by hemodynamic assessment (i.e., ABI ≤ 0.9). The utility of a novel plasma biomarker for PAD would be greatest in individuals who are not suspected to have this disease or underlying risk. A future larger study will be needed to understand if these metabolites alone, or, more likely, a model of these metabolites interacting with other clinical parameters, predict the probability of PAD.

Our observations are largely consistent with what is currently known about these metabolites and atherosclerosis or atherogenesis. IPA, which is a product of the bacterial indole pyruvate pathway of tryptophan metabolism [47], is a ligand for the pregnane X receptor, which is found in many tissues including the endothelium and modulates vasodilation, innate immune receptor expression/function, and endothelial detoxification processes [48,49,50]. We observed an inverse correlation between IPA and advanced atherosclerosis in the previous study [23] and between IPA and claudication in the current study. Similarly, others have found an inverse correlation between circulating IPA and type 2 diabetes mellitus and low-grade inflammation in human population-based studies [51,52]. IPA was also downregulated in patients with coronary artery disease compared to healthy controls, and dietary IPA supplementation attenuated atherosclerotic plaque in a genetic model of atherosclerosis [53].

IS is a uremic toxin formed through hydroxylation of indole in the liver followed by O-sulfation and by the activity of bacterial tryptophanase [47]. IS activates the aryl hydrocarbon receptor (AHR) pathway in primary human aortic vascular smooth muscle cells to promote thrombosis through the upregulation of tissue factor and inhibition of ubiquitination and degradation of tissue factor [54,55]. In endothelial and adipose cells, IS induces proinflammatory cytokines and monocyte/macrophage activation [56]. Serum concentrations of IS are associated with aortic calcification, arterial stiffness, and increased cardiovascular mortality in patients with chronic kidney disease [57]. Among patients with end-stage renal disease on hemodialysis, IS was associated with incident PAD [58].

As with IPA, I3A is a product of TRP metabolism by bacterial tryptophanase [47]. I3A also acts on the AHR pathway [59]. We observed an inverse correlation between I3A and claudication, and elevated I3A reduces risk of MACE. Others have found that I3A increases anti-inflammatory IL-10 receptor expression [60] and reduction in the type I interferon response [61] and inflammatory cytokine profile [62], thus raising the possibility that I3A reduces risk of PAD by attenuating systemic inflammation.

HAA is a downstream product of TRP metabolism to KYN via IDO1 and tryptophan 2,3-dioxygenase followed by conversion of KYN to 3-hydroxykynurenine and then to HAA. IDO1 is induced in the context of inflammation [63] and is involved in autoimmunity, chronic infection, granulomatous diseases, and cancer [64]. In a cohort of patients with stable angina, plasma HAA, in addition to other kynurenines, was associated with risk of acute MI after multivariable adjustment (hazard ratio 1.48; 95% CI 1.10–1.99) and correlated with phenotypes of metabolic syndrome, suggesting that these metabolites could be used to improve risk estimates [65]. In animal models, HAA regulates the inflammasome and decreases plasma lipids and atherosclerosis [66].

We observed a negative correlation between PAD and HA, although the mechanism by which HA impacts cardiovascular disease is unknown. HA, the glycine conjugate of benzoic acid, is part of the endogenous urinary metabolite profile, but levels can be indicative of microbial metabolism of certain nutrients, and it can be a biomarker of toxic compounds such as toluene [67]. In a rat model of diet-induced atherosclerosis, hippurate excretion was higher in the atherosclerosis group compared to the control group, but differences in diets and cage environments were not explored [68]. Notably, hippurate has been shown to correlate with a lean phenotype and a diet rich in flavanols [69,70], which is beneficial for cardiovascular health [71,72,73]. Interestingly, in a large population-based cohort study of patients with hypertension, low urinary hippurate excretion correlated with high blood pressure [74], a finding that is in concordance with the observation that urinary hippurate is lower in spontaneously hypertensive rats compared to normotensive rats [75]. In contrast, in other animal models, elevated hippurate is associated with endothelial dysfunction and accelerated atherosclerosis [76]. Given these controversies, further research is needed to understand the role of HA in cardiovascular disease, either as a biomarker or direct modulator.

While we previously observed that TRP and the KYN/TRP ratio correlated with advanced atherosclerosis [23], we did not observe a significant link between these metabolites and claudication in the current study. However, there was a significant correlation between these metabolites and development of MACE during the follow-up period. As IDO1 activity correlates with systemic inflammation, we surmise that more pronounced baseline inflammation in the previous cohort of patients with advanced atherosclerosis, all of whom underwent either revascularization or amputation, compared to the claudicants in the current cohort, accounts for the differences in metabolomics profiles. Indeed, patients with chronic limb-threatening ischemia are known to have higher circulating inflammatory cytokine levels than patients with claudication [77], and inflammatory markers improve predictive models for adverse events such as major amputation and death in patients with severe limb ischemia [78].

While MACE endpoints are typically adopted for patients with chronic limb-threatening ischemia, claudication is associated with a high risk of cardiovascular morbidity but a low risk of progression of leg symptoms. In a population-based observational study, patients with claudication had 2.6-fold increased risk of cardiovascular death compared to asymptomatic patients [79]. In patients with large-vessel PAD, there was a nearly six-fold relative risk of cardiovascular death over 10 years compared to those without PAD [40]. In a more contemporary prospective cohort, MACE events occurred in 37% and 64% of claudicants at 5 and 10 years, respectively [80]. Thus, we felt that the MACE metric was relevant for this cohort.

This study provides supportive evidence for further investigation of indole- and phenyl-derived microbial metabolites in PAD and, more generally, in cardiovascular disease. We focused solely on claudication, which is a more clinically homogeneous clinical entity than our previous cohort of patients who were a heterogeneous group undergoing revascularization or amputation for advanced atherosclerotic disease in multiple vascular beds. This allowed for better control of potentially confounding factors. Concordance in findings between these two studies argues for assessment of these associations in larger populations to confirm these findings. Since high-density metabolite profiling systems can simultaneously detect microbial metabolites in biofluids (e.g., blood and urine) and tissue biopsies, which can then be integrated with clinical data, it is possible that future studies could provide novel insights into disease-predictive and/or disease-associated microbe-associated biomarkers. Furthermore, given the known crucial roles of many microbial metabolites in host homeostasis and immunology, an in-depth understanding of the genetic control of microbial metabolic pathways and the repercussions of gut microbial community changes to host biochemical and metabolic pathways is of fundamental importance [81,82].

The limitations of this study include its observational nature and inherent potential for unmeasured confounders, such as lack of direct dietary or microbiome data and lack of information on antibiotic exposure and other medications which could impact the microbiome [83,84]. Furthermore, while this mostly male and Caucasian cohort is reflective of the population served by our medical center, its composition potentially limits the generalizability of the findings. Third, a targeted metabolomic approach inherently restricts the panel of candidate markers and focuses on only a few metabolic pathways. However, focused examination of these metabolites arose from a broad untargeted comparative metabolomic analysis of germ-free and conventional mice which identified metabolites that arise exclusively from microbiota or are significantly impacted by microbiota [22]. Finally, although our analysis cannot determine causality, it provides further supportive evidence that alterations in specific indole- and phenyl-derived metabolic species might represent important pathophysiologic mechanisms in PAD that deserve further investigation.

## 5. Conclusions

In summary, the present study identifies specific indole- and phenyl-derived microbial metabolites associated with claudication and MACE. Our findings lay the groundwork for future studies that refine the microbial metabolomic signature of PAD and increase our understanding of how these metabolites could add independent value to existing clinical risk scores for diagnosing and prognosticating clinically relevant outcomes in PAD.

## Figures and Tables

**Table 1:** Baseline characteristics of study population.

Covariates N (%) or Median [Interquartile Range]	Control Cohort (N = 37)	Claudication (N = 119)	*p* Value
Age	67 (60–74)	68 (65–73)	0.19
Male sex	32 (86.5)	115 (96.6)	0.02
African-American	7 (18.9)	23 (19.3)	0.54
Body mass index (kg/m^2^)	29.4 (26.1–31.3)	27.8 (24.4–31.2)	0.18
Coronary artery disease	0	47 (39.5)	<0.0001
Prior coronary revascularization		33 (75%)	
Prior MACE	0	46 (42.4)	<0.0001
Hypertension	25 (67.6)	111 (93.3)	<0.0001
Hyperlipidemia	24 (64.9)	100 (84)	<0.01
Diabetes mellitus	8 (21.6)	41 (34.5)	0.14
Current/former smoker	27 (75)	119 (93.3)	0.002
Aspirin use	19 (51.4)	86 (72.3)	0.02
Statin use	23 (62.2)	95 (79.3)	0.03
ABI	1.1 (1.1–1.2)	0.73 (0.64–0.81)	<0.0001
Total cholesterol, mg/dL	170 (142.5–197)	158 (127–182)	0.10
LDL cholesterol, mg/dL	99 (79.5–124.5)	82 (60–106)	0.008
HDL cholesterol, mg/dL	43.5 (38–47.5)	42 (35–55)	0.89
eGFR, mL/min/1.73 mm^2^	82 (66–107)	70.5 (60.5–86.5)	0.02
hs-CRP, mg/mL	3.3 (1.2–5.1)	2.4 (1.5–6)	0.82
Rutherford classification			<0.0001
0	37 (100)	4 (3.4)	
1	0	38 (32.5)	
2	0	34 (29.1)	
3	0	41 (35.0)	
Metabolites, μmol			
Indole derivatives			
Serotonin	1.3 (.52–2.4)	0.96 (0.48–1.38)	0.02
KYN	3.8 (2.9–5)	2.3 (1.9–4.2)	0.0001
TRP	0.076 (0.047–0.099)	0.052 (0.038–0.076)	0.005
KYN/TRP ratio (x100)	4975 (3717–6783)	4935 (3949–6743)	0.69
IPA	2.7 (1.7–4.1)	1.07 (0.57–1.86)	<0.0001
I3A	0.21 (0.14–0.24)	0.12 (0.10–0.16)	<0.0001
IS	1.3 (0.6–2.4)	2.8 (1.8–4.9)	0.002
HAA	0	0.31 (0.22–0.54)	<0.0001
Phenyl derivatives			
PCS	0.23 (0.13–0.37)	0.31 (0.19–0.51)	0.07
HA	18.4 (11.7–24.2)	9.4 (6.3–16.7)	<0.0001
Cytokines, pg/mL			
IL-6	0.85 (0.63–1.23)	1.2 (0.93–1.81)	0.02
ICAM	206 (172–300)	246 (207–300)	0.06
TNF-α	1.7 (1.3–2.0)	1.98 (1.64–2.35)	0.004
Omega-3 index	0.064 (0.050–0.072)	0.046 (0.040–0.055)	<0.0001

ABI, Ankle–brachial index; eGFR, estimated glomerular filtration rate; HDL, high-density lipoprotein; hs-CRP, high sensitivity C-reactive protein; LDL, low density lipoprotein. All other abbreviations are as described in the text. Red values indicate *p* < 0.05.

**Table 2:** Odds ratios (OR) for claudication obtained from logistic models. In each model, individual metabolites are separately adjusted for sex, smoking, diabetes, hypertension, and hyperlipidemia.

Metabolite	OR	95% CI	*p* Value
ln serotonin	0.69	0.45–1.1	0.1
ln KYN	0.22	0.086–0.57	0.002
ln TRP	0.69	0.34–1.4	0.29
ln KYN/TRP	0.65	0.36–1.15	0.14
ln HA	0.42	0.24–0.72	0.002
ln IPA	0.36	0.21–0.61	0.0002
ln IS	1.8	1.11–3.00	0.02
ln PCS	1.1	0.72–1.72	0.63
ln HAA	4.44	2.6–7.5	<0.0001
ln I3A	0.11	0.03–0.35	0.0002

OR, odds ratio. CI, confidence interval. All other abbreviations are as described in the text. Red values indicate *p* < 0.05.

**Table 3:** Univariate associations with MACE during the follow-up period.

Variable	No MACE N = 111 (79.9%)	MACE N = 28 (20.1%)	*p* Value
Age	67.4 (63.6–73.3)	68.8 (64.4–75.8)	0.35
Male sex	108 (97.3%)	27 (96.4%)	0.99
African-American	24 (21.6%)	4 (14.3%)	0.56
Body mass index (kg/m^2^)	28.6 (25.0–31.2)	27.8 (24.6–32.6)	0.99
Past medical history			
Coronary artery disease	27 (24.3%)	17 (60.7%)	0.0002
Prior coronary revascularization	18 (66.7%)	15 (88.2%)	0.11
Prior MACE	29 (26.1%)	17 (60.7%)	0.0005
Hypertension	98 (88.3%)	25 (89.3%)	0.88
Hyperlipidemia	92 (82.3%)	22 (78.6%)	0.60
Diabetes mellitus	35 (31.5%)	10 (35.7%)	0.67
Current/former smoker	100 (90.9%)	25 (89.3%)	0.79
Aspirin use	76 (68.5%)	20 (71.4%)	0.76
Statin use	82 (73.9%)	23 (82.1%)	0.36
ABI	0.8 (0.7–1.0)	0.7 (0.6–0.8)	0.01
Total cholesterol, mg/dL	160 (136–185)	156 (122–189.5)	0.61
LDL cholesterol, mg/dL	83 (64–110)	83 (57.5–108.5)	0.53
HDL cholesterol, mg/dL	43 (36–54)	45 (35.5–50.5)	0.57
eGFR, mL/min/1.73 mm^2^	73 (63–90)	72 (60–93)	0.76
hs-CRP, mg/mL	2.4 (1.3–5.1)	3.4 (1.7–7.1)	0.28
Rutherford classification			0.08
0	29 (26.4%)	4 (14.3%)	
1	30 (27.3%)	4 (14.3%)	
2	25 (22.7%)	7 (25.0%)	
3	26 (23.6%)	13 (45.4%)	
Metabolites, μmol			
Indole derivatives			
Serotonin	0.96 (0.40–1.49)	1.14 (0.64–1.51)	0.46
KYN	2.84 (2.02–4.57)	2.89 (1.77–3.21)	0.02
TRP	0.06 (0.04–0.09)	0.04 (0.029–0.057)	0.003
KYN/TRP ratio (x100)	4790 (3798–6491)	4955 (4189–6892)	0.30
IPA	1.15 (0.61–2.3)	1.11 (0.63–1.77)	0.65
I3A	0.14 (0.11–0.20)	0.11 (0.096–0.16)	0.02
IS	2.70 (1.55–4.56)	3.03 (1.81–4.40)	0.74
HAA	0.26 (0–0.44)	0.28 (0.11–0.63)	0.32
Phenyl derivatives			
PCS	0.29 (0.17–0.47)	0.32 (0.21–0.70)	0.20
HA	11.0 (7.1–19.7)	9.1 (5.3–16.8)	0.12
Cytokines, pg/mL			
IL-6	1.1 (0.79–1.49)	1.5 (1–2.4)	0.03
ICAM	230 (196.4–280)	272.9 (221.5–308.1)	0.07
TNF-α	1.86 (1.45–2.24)	2.05 (1.82–2.31)	0.04
Omega-3 index	0.07 (0.06–0.08)	0.05 (0.04–0.06)	0.49

CI, confidence interval. All other abbreviations are as described in the text. Red values indicate *p* < 0.05.

## Data Availability

The data used to support the findings of this study are available from the corresponding author upon reasonable request.
